# Evaluating the Role of Circulating Dendritic Cells in Methimazole-Treated Pediatric Graves’ Disease Patients

**DOI:** 10.3390/genes12020164

**Published:** 2021-01-26

**Authors:** Aleksandra Starosz, Karolina Stożek, Marcin Moniuszko, Kamil Grubczak, Artur Bossowski

**Affiliations:** 1 Department of Regenerative Medicine and Immune Regulation, Medical University of Bialystok, 15-269 Bialystok, Poland; aleksandra.starosz@umb.edu.pl (A.S.); marcin.moniuszko@umb.edu.pl (M.M.); 2 Cardiology Unit, Department of Pediatrics, Endocrinology and Diabetes, Medical University of Bialystok, 15-274 Bialystok, Poland; karolina.stozek33@gmail.com

**Keywords:** Graves’ disease, autoimmunity, dendritic cells, methimazole

## Abstract

Graves’ disease (GD) is hyperthyroidism associated with organ-specific autoimmune inflammation. GD occurs more frequently in adults than in children; however, pediatric patients are a therapeutic challenge due to cycles of remissions and relapses requiring constant monitoring at every stage of treatment administered. Dendritic cells (DCs) are considered to be a link between innate and adaptive immunity. DCs, as antigen-presenting cells (APCs), are involved in antigen presentation to T lymphocytes, thereby initiating a shift towards effector cells. In accordance, DCs also participate in the modulation of tolerance to specific antigens. To date, the data on DCs’ role in Graves’ pathological processes are scarce. Therefore, here, we evaluated the frequencies and role of circulating DCs in GD pediatric patients treated with methimazole. Flow cytometric analysis was implemented to evaluate three subsets of dendritic cells and their correlation with clinical GD-related parameters. We found significantly higher levels of DC subsets in patients at diagnosis. Furthermore, methimazole treatment seemed to effectively reduce subsets of DCs, which, in addition, were found to differentially correlate with thyroid function. Our study shed new light on DCs’ role in the pediatric GD pathomechanism. Further studies are required for the mechanistic assessment of DCs’ exact role in disease progression and influence on thyroid function.

## 1. Introduction

Graves’ disease (GD) is the most common cause of hyperthyroidism, a condition associated with abnormal thyroid gland function resulting from an organ-specific autoimmune reaction. Despite local autoaggression of the immune system, affected thyroid function contributes significantly to changes in systemic regulation, leading to, inter alia, weight loss, palpitation, hyperactivity, and orbitopathy [1,2]. In children, GD occurs less frequently compared to adults; however, it remains a severe therapeutic problem and requires constant monitoring due to alternate periods of remissions and relapses [3]. Although the etiology of GD is still not fully understood, the classical paradigm involves a role of genetic, environmental, and immune-related factors [4,5]. Abnormalities in immune system function lead to uncontrolled production of thyroid-stimulating hormone receptor (TSHR) autoantibodies (TRAb), thus inducing excessive thyroid hormone synthesis and consequently resulting in hyperthyroidism [6]. Moreover, previous research indicates that increased activity of T lymphocytes, predominantly helper Th1 and Th2, is responsible for the inflammation phenomenon occurring in GD [7]. To date, participation of other populations of immune cells has been demonstrated by numerous papers, including effector Th17, regulatory T lymphocytes (Treg), and even tissue-resident fibroblasts [8,9]. However, to date, there are no comprehensive data on the role of dendritic cells in the course of pediatric Graves’ disease.

Dendritic cells (DCs) are a heterogeneous population of cells originating from the bone marrow, classified as highly specialized antigen-presenting cells (APCs). Their primary role is to induce and regulate the immune response through effector cells’ activation. Dendritic cells are also considered to be a link between the innate and acquired immune response, which is due to the fact that they recognize pathogens by receptors such as Toll-like receptors (TLRs) belonging to non-specific immunity. Apart from pathogen presentation to T-lymphocytes and inducing effector phenotypes, DCs modulate response of T lymphocytes to specific antigens in the body, thus controlling tolerance mechanisms [10,11,12]. Furthermore, DCs are thought to be involved in T lymphocyte plasticity through controlling Th1 and Th2 differentiation [1,13,14].

Presently, two major subpopulations of dendritic cells can be distinguished: plasmacytoid DCs (pDC), indicated by the presence of the CD303 marker, and myeloid/classical DCs (mDC/cDC). Additionally, myeloid DCs can be further divided into two subgroups: mDC1 marked by CD1c, and mDC2 recognized by the CD141 marker [10]. Plasmacytoid dendritic cells (pDCs), immediately after contact with an antigen, produce mainly type I interferons (IFNα) responsible for modulating the activity of immune cells by increased TLR expression induction. Effects caused by pDC also include an increase in the production of proinflammatory cytokines and chemokines. Furthermore, pDC can stimulate the differentiation of autoreactive Th naïve (Th0) cells into effector T cells, especially Th2, releasing inflammatory cytokines [15,16]. On the contrary, myeloid dendritic cells are involved predominantly in presenting antigens to T cells, as they demonstrate higher levels of MHC class II molecules [10,17]. mDCs1 are mainly engaged in antibacterial and antifungal immune responses due to the increased expression of MHC class I and II molecules, thus promoting the activation of CD4+ and CD8+ lymphocytes and secretion of IFN-γ by these effector cells. Moreover, mDC1 express TLR-2 and scavenger receptors responsible for the instant recognition of antigens and modulation of the immune response [18]. In turn, mDCs2 can be recognized by a high expression of TLR-3, secretion of IL-12 and IFN-β, and ability to induce T cell responses, especially Th1 [19]. Dendritic cells can contribute to the initiation of tolerance mechanisms in various ways, but therefore, they can also play a role in immune tolerance impairment, which is one of the causes of autoimmune disease onset. Accordingly, there is a gradually increasing number of the reports on the alleged involvement of dendritic cells in autoimmune diseases by affecting tolerance mechanisms [20,21,22].

Considering scarce data on dendritic cells in pediatric Graves’ disease, which in fact are involved in tolerance induction counteracting the development of autoimmune diseases, our research aimed to assess the changes in dendritic cells in non-thyroid treated children with the disease at diagnosis. Moreover, here, we evaluated how DC subsets correlate with thyroid function-related parameters and the influence of methimazole (MMI) treatment on these dependencies.

## 2. Materials and Methods

### 2.1. Patients

The study was performed on 22 pediatric patients with active Graves’ disease (GD). The additional control group (HC) involved 31 healthy patients with autoimmune and inflammatory conditions excluded—euthyroid and no personal or family history of autoimmune thyroid disease (AITD). The clinical description of the patient groups is included in Table 1 (Table 1). Informed consent was obtained from each of the patients (parent or legal guardian for underage subjects). The protocol of the study was approved by the Ethical Committee at the Medical University of Bialystok (R-I-002/422/2010).

The research material was EDTA-anticoagulated peripheral blood collected by venipuncture at three time points: before treatment (T0) and after 3 months (T1) and 1 year of treatment (T2) with methimazole from pediatric patients. Peripheral blood mononuclear cells (PBMCs) were isolated through gradient centrifugation using Pancoll with a density of 1.077g/l (PAN Biotech, Aidenbach, Germany). Cells were washed and suspended in cryoprotectant 10% DMSO (Sigma-Aldrich, St. Louis, MO, USA) in fetal bovine serum (FBS, Belize, CA, USA; PAN Biotech) and were stored as viable cells in liquid nitrogen until the described experiments.

### 2.2. Flow Cytometry

Following thawing of PBMCs from liquid nitrogen, cell counting, and viability verification, cells were subjected to flow cytometric analysis. PBMCs of Graves’ disease patients (GD) and the control group (HC) were stained with monoclonal antibodies conjugated to fluorochromes aimed at selected cell surface markers allowing for dendritic cells analysis, including anti-Lineage2 (anti-CD3 FITC, clone SK7; anti-CD14 FITC, clone MφP9; anti-CD19 FITC, clone SJ25C1; anti-CD20 FITC, clone L27; anti-CD56 FITC, clone NCAM16.2), anti-CD1c AlexaFluor647 (clone F10/21A3), anti-CD141 PE (clone 1A4) (BD Bioscience, Franklin Lakes, NJ, USA), and anti-CD303 PE-Cy7 (clone 201A) (Biolegend, San Diego, CA, USA). Equal amounts of PBMCs were stained in the same buffer volume for all of the patients to investigate absolute numbers of cells. Following staining of cells and washing unbound antibodies in phosphate-buffered saline (PBS, Arlington County, VA, USA; Corning, Corning, NY, USA), cells were fixed with the use of CellFix reagent (CellFix, Fresno, CA, USA; BD Bioscience) and stored shortly until the acquisition at +4 °C. Data were acquired on a FACS Calibur flow cytometer (BD Bioscience) and subsequently analyzed using FlowJo software (Tree Star Inc., Ashland, OR, USA).

Dendritic cells were distinguished based on morphological properties (relative size—forward scatter (FSC), and relative shape/granularity—side scatter (SSC)) and lack of cell surface markers included in the Lineage2 reagents: CD3, CD14, CD19, CD20, and CD56. Furthermore, stratification of dendritic cells was possible by evaluating the differential expression of CD1c, CD141, and CD303 cell surface markers. Accordingly, we were able to monitor changes in plasmacytoid DCs (CD303+, pDC) and two subtypes of myeloid/classical DCs (CD141+, cDC2; CD1c+, cDC1). In addition, various combinations of selected markers were included in the analysis of DCs: CD1c+CD141+, CD1c+CD303+, CD141+CD303+, and CD1c+CD141+, CD303+). The implemented gating strategy was included in the Supplementary Materials (Figure S1).

### 2.3. Statistical Analysis

Biostatistical analysis of the obtained flow cytometric data was performed with the use of GraphPad Prism 9.0.0 statistical software (GraphPad Prism Inc., San Diego, CA, USA). Considering the lack of Gaussian distribution of the data within studied groups, two-way ANOVA tests were applied with the Sidak correction. The test for paired data was used for assessment of changes in Graves’ disease patients in the course of treatment, and that for unpaired data when comparing Graves’ disease patients to the control group. The evaluation of correlations between the studied dendritic cell-related parameters and clinical results was performed using the Spearman test. The final results were presented on graphs as the frequency of studied markers within Lineage2-negative PBMC cells, frequency of Lineage2-negative cells with selected marker within total PBMC cells, and as an absolute number of cells.

## 3. Results

### 3.1. Graves’ Disease Is Associated with Higher Numbers of Circulating Dendritic Cells Compared to Healthy Control Groups

**Figure 1:**
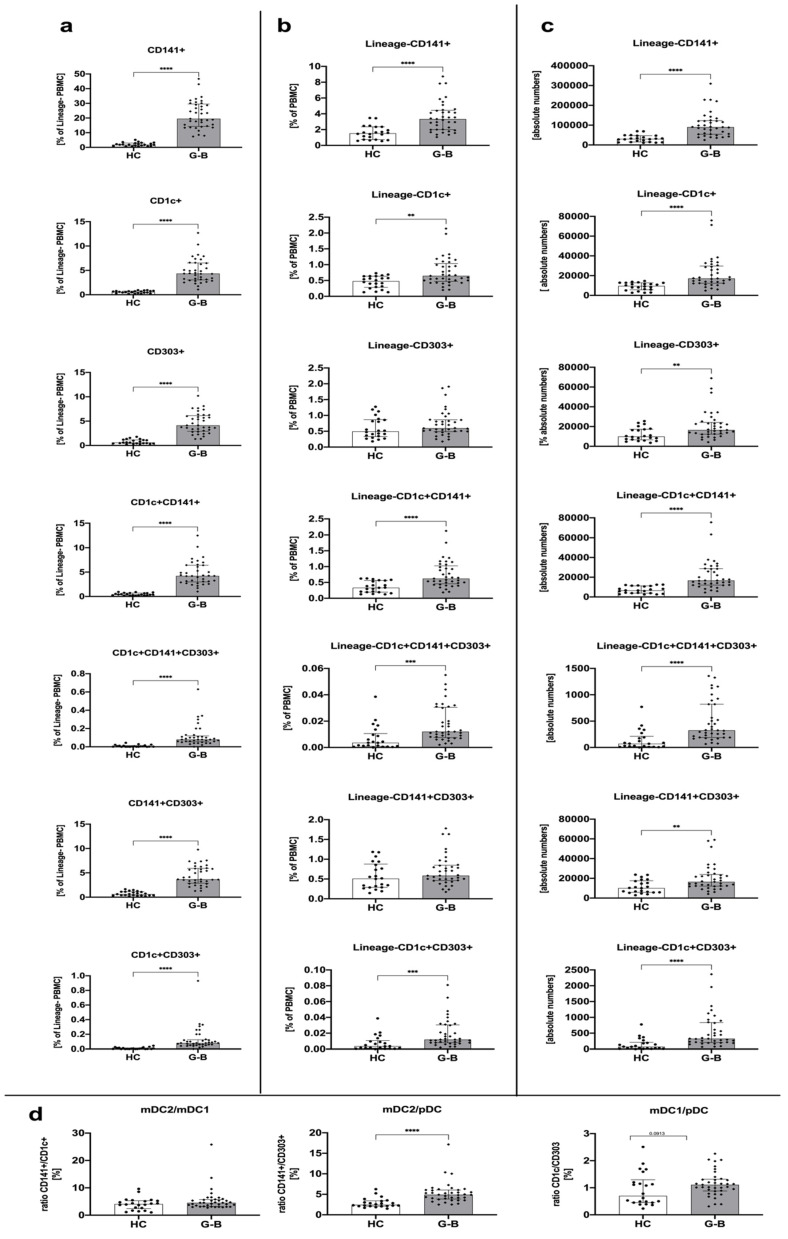
Dendritic cell-related differences among Graves’ disease patients (GD) and the healthy control group (HC). Acquired data were analyzed in the context of dendritic cell (DC) subset frequency within Lineage-negative peripheral blood mononuclear cells (PBMCs) (**a**), the total pool of PBMCs (**b**), as an absolute number of cells (**c**), and the ratio between studied DCs (**d**). Data are presented on each graph as the median with interquartile range. The levels of significant differences were indicated with: ** *p* < 0.01; *** *p* < 0.001; **** *p* < 0.0001.

We found that untreated Graves’ disease patients demonstrated significantly higher levels of dendritic cells compared to the healthy control group. These differences were observed in all studied subtypes of dendritic cells, CD141- and CD1c-expressing myeloid mDCs, and CD303+ plasmacytoid pDCs, especially when frequency within Lineage-negative PBMCs or absolute numbers were analyzed. Although the CD141+ subtype of dendritic cells was the dominant population among others in the context of absolute cell numbers, the ratio of changes between Graves’ disease patients and the control group seemed to be similar among all monitored DC-related parameters (Figure 1a–c). Interestingly, we found a significant difference in ratio between mDC2 and pDC with a strong significance level. Such a phenomenon was not found in mDC2/mDC1, with only a slight tendency in the mDC1/pDC ratio (Figure 1d).

### 3.2. Treatment with Methimazole Influences Changes in Dendritic Cell Distribution in Graves’ Disease Pediatric Patients

**Figure 2:**
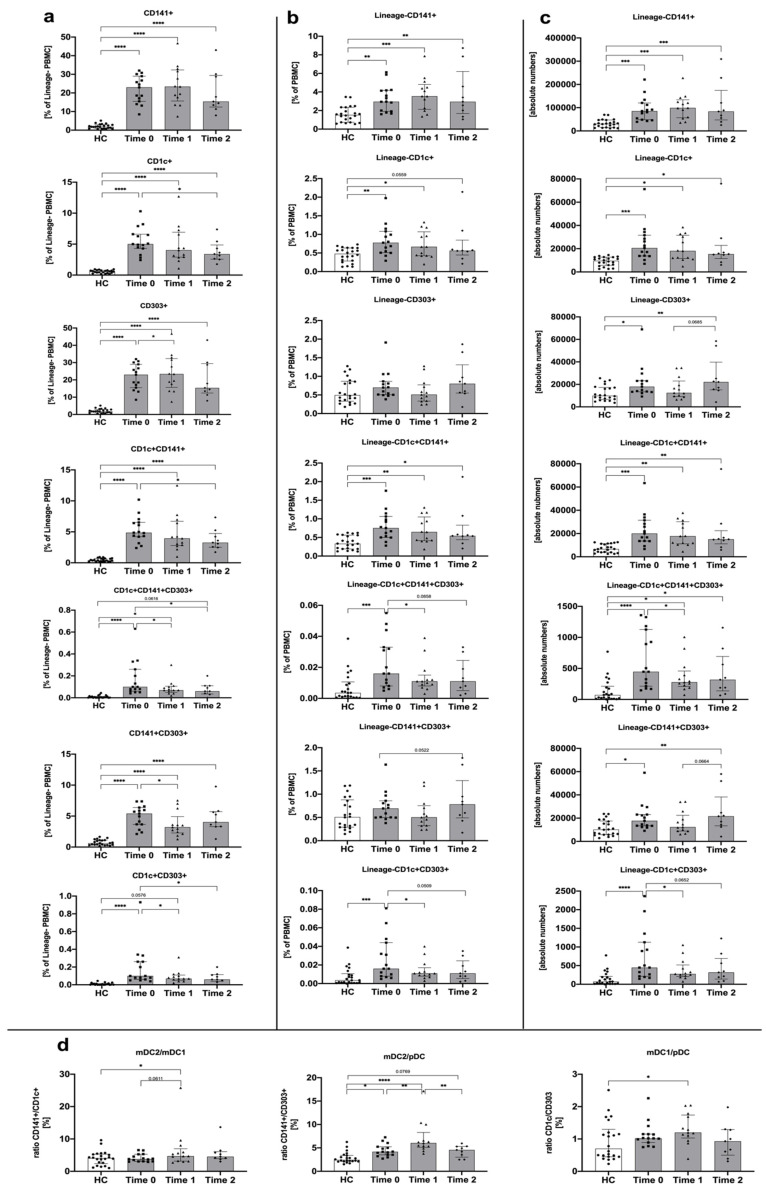
Changes in dendritic cell-related parameters in the course of Graves’ disease patients’ treatment: before treatment (Time 0) and after 3 months (Time 1) and 1 year of treatment (Time 2). Acquired data were analyzed in the context of DC subset frequency within Lineage-negative PBMCs (**a**), the total pool of PBMCs (**b**), as an absolute number of cells (**c**), and the ratio between studied DCs (**d**). Data are presented on each graph as the median with interquartile range. The levels of significant differences were indicated with: * *p* < 0.05; ** *p* < 0.01; *** *p* < 0.001; **** *p* < 0.0001.

We found that methimazole implementation in Graves’ disease patients did not lead to general significant changes in all DC subsets within the first 3 months of the therapy. An exception was subtypes of DC with surface expression of the CD303 marker characteristic for plasmacytoid DCs, even in combination with surface expression of CD1c and/or CD141 typical for myeloid DCs. Reduction of these populations over the course of treatment was reported both at frequency and absolute number levels. In addition, such direction of changes was maintained until further time points were reported, especially in reference to CD1c+CD303+ and CD1c+CD141+CD303+ dendritic cells. Similarly, a slight change was also observed in the CD303+ pDCs analyzed as the frequency within Lineage- PBMCs. Noteworthy, considering mDC-related markers, CD1c+ mDC1s seemed to respond to the methimazole regimen with a significant decline in frequency and number of these cells after 1 year of therapy. No differences were found in dendritic cells expressing the CD141 marker (Figure 2a–c).

Furthermore, ratio analysis indicated that changes in myeloid mDC2 and plasmacytoid pDC are more profound over the course of treatment with methimazole. Moreover, a higher decline in pDCs than mDC2s might be responsible for the increased ratio of mDC2/pDC after 3 months of therapy. In accordance, the less significantly increased mDC1/pDC ratio at the third month of methimazole application suggests a higher role of changes in CD141+ mDC2 and CD303+ pDC over the course of Graves’ disease management (Figure 2d).

### 3.3. Dendritic Cells Are Demonstrated to Correlate with Disease-Related Clinical Parameters in a Treatment-Dependent Manner

**Figure 3:**
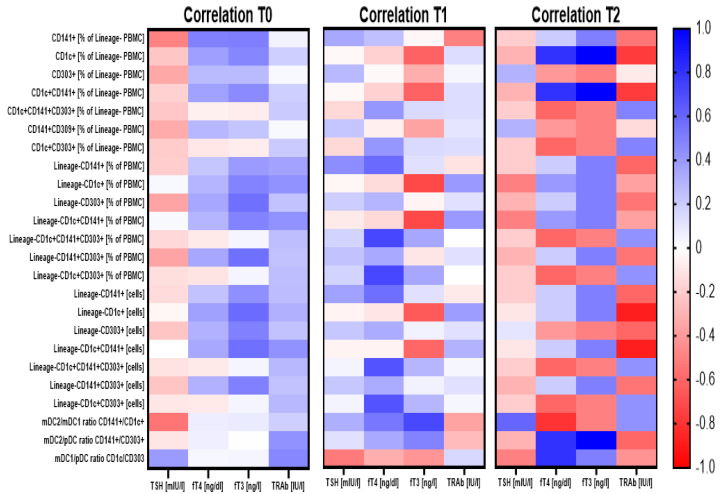
Demonstration of correlations between dendritic cell-related parameters and critical clinical analysis results prior to and during the treatment course. Correlation analysis demonstrated for three time points for Graves’ disease pediatric patients: before treatment (T0) and after 3 months (T1) and 1 year (T2) of the treatment. Strength of correlations (r value) are demonstrated through color gradient—blue for positive correlations and red for negative correlations.

Before the beginning of methimazole treatment, analyzed subtypes of dendritic cells were found to correlate with TSH levels negatively, and on the contrary, a positive correlation was found in reference to fT4, fT3, and TRAb. It is worth noting that none of the population exhibited dominance at that time point in their association with clinical parameters. Despite the relatively sustainable phenomenon observed before treatment, 3 months of methimazole seemed to cause critical changes within dendritic cell populations. CD303+ pDC and CD141+ mDC2 subtypes only correlated with TSH, and importantly, unlike at time 0, here, a positive link was found. In addition, a subtype of CD1c+ mDC1 was demonstrated to change its role in reference to fT3 levels with a shift from positive to negative correlation at the third month of therapy. Following 1 year of treatment, correlations between DC-related parameters and clinical data were comparable to those observed before therapy implementation. However, contrary to the positive correlation of DCs with TRAb at time 0, here, changes within dendritic cells seemed to be associated with the completely different response of TRAbs. Similarly, the correlation of the CD303+ pDC subtype with clinical data was affected over the course of treatment, as that population was the only among others demonstrating a negative correlation with fT4 and fT3 levels (Figure 3; Table S1).

## 4. Discussion

Graves’ disease is characterized by disturbances in immune tolerance. Reactive autoantibodies, especially TSHR antibodies (TRAb), influence the course and the clinical manifestation of the disease [23]. Dendritic cells can create tolerance mechanisms in various ways, while lack of self-tolerance is one of the causes of autoimmune processes. Accordingly, an increasing number of studies on DCs assume their potential contribution to autoimmune diseases by modifying tolerance together with lymphocytes. [24]. The mechanisms of dendritic cells’ participation in autoimmune processes does not seem to be associated only with changes in the distribution of circulating and tissue-resident dendritic cells. Notably, impairment of DC function may result in the breakdown of self-tolerance, leading to autoimmune disorders [10]. Dendritic cells’ contribution to the autoimmunity processes occurs through two main cellular mechanisms. On one hand, DCs are responsible for maintaining immune tolerance (immunosuppression effect) via induction of the regulatory T cell phenotype through differentiation from CD4+ naïve T lymphocytes, associated inter alia with higher production of TGF-β. On the other hand, dendritic cells can promote adaptive self-reactive responses and thus cause loss of tolerance. Lack of response to the antigen also affects the inhibition of T cell activity, especially CD8+ cytotoxic phenotypes [25]. Rönnblom et al. proved that pDCs may induce Th1-related reactions, produce type I INF, and thereby lead to the autoimmune phenomenon in the pathogenesis of systemic lupus erythematosus [26]. Moreover, DCs’ ability to present the antigen (APCs), here in the context of autoimmune disorders and autoantigens, might be a feature responsible for the induction of effector cells that consequently acquire autoreactive status. Noteworthily, despite numerous cases of partial information on DCs in autoimmunity, there are only a few studies aimed directly at the DCs’ role in GD. To date, our study is the first research that focuses on a pediatric population of Graves’ disease in the context of dendritic cells and their relation to the treatment effects.

In our research, we found a significant increase in the number of dendritic cells in GD patients compared to the healthy control group (Figure 1). The demonstrated increased percentage of circulating DCs might be directly related to the allegedly increased proportion of DCs involved in antigen presentation, and thus the induction of lymphocytes producing anti-TSH-R autoantibodies. The dominant subpopulation of DCs presenting antigens to lymphocytes is plasmacytoid DCs, characterized by the presence of the CD303 molecule on their surface [27]. In our GD patients, we found significantly higher levels of CD303-positive DCs cells at the time of admission compared to the control group. Furthermore, a slight decrease in the frequency of CD303+ DCs over the course of treatment suggests that methimazole can effectively reduce the percentage of the pDC population. Presumably, such a decline in pDCs might be associated with better outcome of the treatment, which can be supported by the fact that a negative correlation was found between the number of CD303-positive DCs and the TSH level (thyrotropic hormone). Noteworthily, in accordance with the link between pDC and TSH levels, a tendency for positive correlation was found between that population and fT3 and fT4 levels. Therefore, the reduction of pDCs is against the background of the MMI treatment might also be associated with lower levels of fT3 and fT4. In addition, the mechanism of thyroid function restoration did not seem to be associated directly with tolerance induction, as we did not find any correlation between CD303-positive dendritic cells and the level of anti-TSH-R autoantibodies (TRAb).

As indicated above, the data on DCs’ role in Graves’ disease are scarce; however, increased numbers of pDCs were reported at the onset of other autoimmune diseases, including type 1 diabetes mellitus [24]. In a non-obese diabetic mice model, the presence of a special DC phenotype, CD103+, was associated with the occurrence of pancreatic islet-reactive CD4+ T cells initiating an autoimmune cascade. Interestingly, ablation of these CD103+ DC genes led consequently to proper immune tolerance and a lack of diabetes-related autoimmune features [28]. Similarly, to diabetes, in the dermal autoimmune disorder psoriasis, an increase of DCs was detected in the epidermis and dermis of affected patients [29].

It is worth noting that plasmacytoid dendritic cells (pDC), by the secretion of interferons, predominantly type I interferons, can induce the maturation of myeloid DC into fully active mDCs. Such a mechanism could explain autoimmune inflammation in systemic lupus erythematosus, where pDC-related production of type 1 interferons caused mDC maturation and consequently their increased interaction with the lymphocytes [30]. Mature mDCs can interact with CD4+ lymphocytes, triggering inflammatory activity of Th1 and Th17 subtypes and enhancing the activity of cytotoxic CD8+ lymphocytes. Additionally, some data suggest that mutual interactions between mDC and B lymphocytes might be responsible for the induction of autoantibodies’ secretion [31,32].

Myeloid/classical dendritic cells, comprised of mDC1 (CD1c+) and mDC2 (CD141+) subsets, are predominantly responsible for presenting antigens to T cells [21]. Here, we observed statistically significant differences in mDCs in patients with GD compared to controls. Furthermore, methimazole treatment led to a slight decrease in mDC1 and mDC2 subsets. Interestingly, methimazole allowed to stabilize the ratio of both mDC1/pDC and mDC2/pDC, causing their gradual decrease over 3 months to a year after therapy initiation. This value shows a downward trend to reach the level observed in the control group (HC). Noteworthily, we did not observe such a type of changes in the context of the mDC2/mDC1 ratio during the whole period of treatment. Only a slight tendency for an increase was demonstrated at the third month of methimazole treatment. In general, reported differences are very interesting in the context of significant phenotype shifts between dendritic cells subsets, which in fact contribute differently to the phenomena occurring in the Graves’ disease progression. In addition to mDC-related events, a strong positive correlation of CD141+CD1c+ DC population frequency with fT3 and fT4 levels observed after one year of treatment might indicate their potential use as markers of favorable control of the disease with methimazole treatment.

Against the background of methimazole (MMI) treatment, we found that applied therapy significantly affects the studied populations of DCs. Implementation within the first 3 months of treatment did not lead to crucial changes within all dendritic cell subsets, but subsets demonstrating CD303 expression (pDCs) showed a decrease in their number. Treatment extended over 3 months contributed to a significant decline in mDC2 and pDC number, with higher decrease observed in pDCs than mDC2, which probably led to the increased ratio of mDC2/pDC. A less pronounced increase in the mDC1/pDC ratio at the third month of methimazole treatment suggests a more essential role of the shifts between CD141+ mDC2 and CD303+ pDC than CD1c+ mDC1 over the course of Graves’ disease management. Effects of prolonged treatment with antithyroid drugs and changes in levels of DCs might indicate an immunomodulatory role of methimazole. MMI is often postulated as a therapeutic with direct immunosuppressive facilities, according to the number and functions of various types of immunocompetent cells in peripheral blood [33]. It has been proven that methimazole reduces the serum levels of cytokines and may influence a shift from Th1 towards Th2 responses in GD [34,35]. Crescioli et al. for the first time evidenced that MMI decreases CXCL10 protein in thyrocytes, which is involved in Th1 immune responses [36]. Bossowski et al. took upon the topic of effector T lymphocytes, predominantly Th17, in autoimmune thyroid diseases. They demonstrated a significantly lower frequency of cells with the CD4+IL17+ phenotype in newly diagnosed GD and its normalization after 2–3 months of methimazole therapy [37]. Here, we demonstrated for the first time that methimazole’s properties also significantly affect the phenotype of dendritic cells and shifts between the studied subsets. The mechanism leading to the observed effects requires further investigation; however, considering the influence of Graves’ disease therapy on adhesion molecules, one of the possible explanations arises. Adhesion molecules such as VCAM-1 and ICAM-1 are markedly elevated in the sera of patients with GD, and importantly, decline after treatment [38,39]. In accordance, the decreased number of DCs occurring in response to GD therapy might be associated with changes in these molecules and consequently affected migration.

Regarding the relationship between DCs and thyroid hormone levels before and after MMI treatment, we found a positive correlation between all analyzed subtypes of DCs versus fT4, fT3, and TRAb levels, with a concomitantly negative correlation of CD303+ pDCs with TSH concentration. As commonly observed, elevated levels of fT4, fT3, and TRAb and low concentrations of TSH were detected in the studied group of pediatric GD patients at the time of admission. In the context of high levels of DC subsets before treatment, that phenomenon could be partially explained by fT3 concentrations. Levels of fT3 were found to affect the maturation of peripheral blood monocytes into functional DCs, and further, their activation and changes in the expression of MHC II and costimulatory molecules [16,40,41,42]. Noteworthily, we demonstrated a link between, inter alia, fT3 and DCs; thus, the reduction of these cells over the course of methimazole treatment must at least to a certain degree be associated with that hormone.

Cumulatively, pediatric patients with GD may demonstrate various deficiencies in their immunological system. Circulating antibodies and infiltration of autoreactive lymphocytes initiate the cascade of autoimmunization. DCs classified as immunogenic or tolerogenic exhibit a dual nature, activating either autoreactive effectors or regulatory T cells [43,44,45]. Here, we demonstrated that Graves’ disease in children is associated with increased frequencies of circulating dendritic cells. Interestingly, apart from previously reported effects, we found that also in the context of DCs, methimazole can influence changes in these cells. Moreover, not only declines in DC levels were reported, but also shifts between myeloid or plasmacytoid DC phenotypes in response to the therapy. However, it is worth noting that the direct role of dendritic cells in the etiology of AITD remains uncertain, and thus further investigations are required for better determination of the mechanism behind changes in DCs over the course of Graves’ disease.

## 5. Conclusions

Disturbances in immune tolerance are one of the main causes leading to the development of autoimmune diseases. Considering partial information indicating the potential involvement of dendritic cells in autoimmune diseases, studies on patients with Graves’ disease are of great importance and might contribute significantly to scientific knowledge in that field. In our study, for the first time, we found that pediatric GD is associated with significantly higher levels of three main subsets of DCs. Moreover, we showed crucial correlations of these cells with thyroid function-related parameters, and in addition, favorable effects of methimazole on the observed phenomenon. We believe that further comprehensive research concerning DCs in pathogenesis GD, and likewise, their individual subsets, might allow the establishment of the direct role of DCs in disease progression and response to treatment, and thus establish novel potential biomarkers or targets for immunotherapeutic development.

## Figures and Tables

**Table 1:** Clinical description of the studied groups Graves’ disease patients (GD) and the healthy control group (HC). Graves’ disease patients were classified on the course of treatment: before treatment (Time 0), after 3 months (Time 1) and 1 year of treatment (Time 2). Data presented as median values with 25th and 75th percentile in the brackets. The significant differences were evaluated compared to the control group (** *p* < 0.01; *** *p* < 0.001).

Parameter	Graves’ Disease (GD) Time 0	Graves’ Disease (GD) Time 1	Graves’ Disease (GD) Time 2	Control Group (HC)
Age (years)	14 (10.75; 16.00)			13.5 (9.50; 14.50)
Sex distribution (male to female)	18% to 82% 4 to 18			39% to 61% 12 to 19
TSH (mIU/L) Ref. range: 0.32–5.0	0.02 *** (0.01; 0.48)	0.23 ** (0.04; 1.89)	1.56 (0.78; 15.21)	2.39 (1.40; 2.80)
fT4 (ng/dL) Ref. range: 0.71–1.55	7.77 (2.28; 7.77)	1.08 (0.42; 1.81)	1.21 (0.41; 1.50)	
fT3 (ng/L) Ref. range: 2.6–5.4	24.59 (5.31; 32.55)	3.92 (2.15; 5.58)	3.52 (1.86; 6.26)	
TRAb (IU/L) Ref. range: 0–1.7	27.90 (15.20; 36.77)	11.39 (3.60; 24.28)	6.46 (2.42; 11.28)	

## Data Availability

Not applicable.

## Supplementary Materials

The following are available online at https://www.mdpi.com/2073-4425/12/2/164/s1, Figure S1: Gating strategy for circulating dendritic cells analysis in Graves patients (FMO controls presented as histograms with dashed lines and no coloring). Table S1: Association between dentritic cell-related data and clinical parameters at three time points: before treatment (T0), after 3 months (T1) and 1 year of treatment (T2).
