# Supplementary material for: Evaluating the Role of Circulating Dendritic Cells in Methimazole-Treated Pediatric Graves’ Disease Patients
Source: Genes (Basel). 2021 Jan 26;12(2):164. doi: 10.3390/genes12020164 (PMC7911035; doi:10.3390/genes12020164)
Supplement: Supplementary file 1 [file genes-12-00164-s001.zip › supplementary/Supp. Tab. 1.docx]

| **Before treatment (T0)** | **TSH [mIU/L]** | | **fT4 [ng/dL]** | | **fT3 [ng/L]** | | **TRAb [IU/L]** | |
| --- | --- | --- | --- | --- | --- | --- | --- | --- |
|  | **r** | **p** | **r** | **p** | **r** | **p** | **r** | **p** |
| \| **CD141+ [% of Lin2- PBMC]** \| \| --- \| \| **CD1c+ [% of Lin2- PBMC]** \| \| **CD303+ [% of Lin2- PBMC]** \| \| **CD1c+CD141+ [% of Lin2- PBMC]** \| \| **CD1c+CD141+CD303+ [% of Lin2- PBMC]** \| \| **CD141+CD303+ [% of Lineage- PBMC]** \| \| **CD1c+CD303+ [% of Lin2- PBMC]** \| \| **Lin2-CD141+ [% of PBMC]** \| \| **Lin2-CD1c+ [% of PBMC]** \| \| **Lin2-CD303+ [% of PBMC]** \| \| **Lin2-CD1c+CD141+ [% of PBMC]** \| \| **Lin2-CD1c+CD141+CD303+ [% of PBMC]** \| \| **Lin2-CD141+CD303+ [% of PBMC]** \| \| **Lin2-CD1c+CD303+ [% of PBMC]** \| \| **Lin2-CD141+ [cells]** \| \| **Lin2-CD1c+ [cells]** \| \| **Lin2-CD303+ [cells]** \| \| **Lin2-CD1c+CD141+ [cells]** \| \| **Lin2-CD1c+CD141+CD303+ [cells]** \| \| **Lin2-CD141+CD303+ [cells]** \| \| **Lin2-CD1c+CD303+ [cells]** \| \| **mDC2/mDC1 ratio CD141+/CD1c+** \| \| **mDC2/pDC ratio CD141+/CD303+** \| \| **mDC1/pDC ratio CD1c/CD303** \| | \| **-0,490** \| \| --- \| \| -0,224 \| \| -0,343 \| \| -0,180 \| \| -0,222 \| \| -0,329 \| \| -0,204 \| \| -0,196 \| \| 0,025 \| \| -0,357 \| \| 0,025 \| \| -0,150 \| \| -0,357 \| \| -0,131 \| \| -0,144 \| \| -0,038 \| \| -0,231 \| \| 0,000 \| \| -0,113 \| \| -0,231 \| \| -0,095 \| \| **-0,542** \| \| -0,098 \| \| 0,394 \| | \| **0,077** \| \| --- \| \| 0,436 \| \| 0,226 \| \| 0,531 \| \| 0,439 \| \| 0,246 \| \| 0,478 \| \| 0,495 \| \| 0,933 \| \| 0,208 \| \| 0,933 \| \| 0,603 \| \| 0,208 \| \| 0,649 \| \| 0,619 \| \| 0,895 \| \| 0,421 \| \| 1,000 \| \| 0,695 \| \| 0,421 \| \| 0,743 \| \| **0,047** \| \| 0,735 \| \| 0,161 \| | \| **0,491** \| \| --- \| \| 0,375 \| \| 0,268 \| \| 0,366 \| \| -0,062 \| \| 0,280 \| \| -0,096 \| \| 0,222 \| \| 0,288 \| \| 0,344 \| \| 0,288 \| \| -0,085 \| \| 0,344 \| \| -0,107 \| \| 0,237 \| \| 0,368 \| \| 0,304 \| \| 0,337 \| \| -0,083 \| \| 0,304 \| \| -0,087 \| \| 0,066 \| \| 0,062 \| \| 0,027 \| | \| **0,076** \| \| --- \| \| 0,184 \| \| 0,349 \| \| 0,196 \| \| 0,830 \| \| 0,328 \| \| 0,740 \| \| 0,441 \| \| 0,313 \| \| 0,226 \| \| 0,313 \| \| 0,768 \| \| 0,226 \| \| 0,710 \| \| 0,409 \| \| 0,193 \| \| 0,287 \| \| 0,235 \| \| 0,774 \| \| 0,287 \| \| 0,762 \| \| 0,820 \| \| 0,832 \| \| 0,924 \| | \| **0,504** \| \| --- \| \| 0,472 \| \| 0,269 \| \| 0,451 \| \| -0,074 \| \| 0,231 \| \| -0,074 \| \| 0,395 \| \| 0,483 \| \| **0,560** \| \| 0,483 \| \| 0,036 \| \| **0,560** \| \| 0,036 \| \| 0,437 \| \| **0,577** \| \| 0,490 \| \| **0,556** \| \| 0,036 \| \| 0,490 \| \| 0,036 \| \| 0,077 \| \| -0,007 \| \| 0,036 \| | \| **0,096** \| \| --- \| \| 0,122 \| \| 0,393 \| \| 0,142 \| \| 0,14159363142002 \| \| 0,817 \| \| 0,466 \| \| 0,817 \| \| 0,202 \| \| 0,113 \| \| **0,061** \| \| 0,113 \| \| 0,909 \| \| **0,061** \| \| 0,909 \| \| 0,155 \| \| **0,052** \| \| 0,107 \| \| **0,063** \| \| 0,909 \| \| 0,107 \| \| 0,909 \| \| 0,812 \| \| 0,986 \| \| 0,909 \| | \| 0,047 \| \| --- \| \| 0,190 \| \| 0,023 \| \| 0,190 \| \| 0,204 \| \| 0,023 \| \| 0,204 \| \| 0,357 \| \| 0,428 \| \| 0,238 \| \| 0,428 \| \| 0,251 \| \| 0,238 \| \| 0,251 \| \| 0,261 \| \| 0,309 \| \| 0,238 \| \| 0,428 \| \| 0,275 \| \| 0,238 \| \| 0,275 \| \| 0,190 \| \| 0,428 \| \| 0,469 \| | \| 0,934 \| \| --- \| \| 0,664 \| \| 0,976 \| \| 0,664 \| \| 0,626 \| \| 0,976 \| \| 0,626 \| \| 0,389 \| \| 0,299 \| \| 0,582 \| \| 0,299 \| \| 0,548 \| \| 0,582 \| \| 0,548 \| \| 0,536 \| \| 0,461 \| \| 0,582 \| \| 0,299 \| \| 0,507 \| \| 0,582 \| \| 0,507 \| \| 0,664 \| \| 0,299 \| \| 0,243 \| |

**Supp. Tab. 1** Tabular presentation of the correlation results. Association between dentritic cell-related data and clinical parameters at three time points: before treatment (T0), after 3 months (T1) and 1 year of treatment (T2). Data presented as correlation coefficient (r) and statistical significance p-value (p).

| **3 months of treatment (T1)** | **TSH [mIU/l]** | | **fT4 [ng/dl]** | | **fT3 [ng/l]** | | **TRAb [IU/l]** | |
| --- | --- | --- | --- | --- | --- | --- | --- | --- |
|  | **r** | **p** | **r** | **p** | **r** | **p** | **r** | **p** |
| \| **CD141+ [% of Lin2- PBMC]** \| \| --- \| \| **CD1c+ [% of Lin2- PBMC]** \| \| **CD303+ [% of Lin2- PBMC]** \| \| **CD1c+CD141+ [% of Lin2- PBMC]** \| \| **CD1c+CD141+CD303+ [% of Lin2- PBMC]** \| \| **CD141+CD303+ [% of Lineage- PBMC]** \| \| **CD1c+CD303+ [% of Lin2- PBMC]** \| \| **Lin2-CD141+ [% of PBMC]** \| \| **Lin2-CD1c+ [% of PBMC]** \| \| **Lin2-CD303+ [% of PBMC]** \| \| **Lin2-CD1c+CD141+ [% of PBMC]** \| \| **Lin2-CD1c+CD141+CD303+ [% of PBMC]** \| \| **Lin2-CD141+CD303+ [% of PBMC]** \| \| **Lin2-CD1c+CD303+ [% of PBMC]** \| \| **Lin2-CD141+ [cells]** \| \| **Lin2-CD1c+ [cells]** \| \| **Lin2-CD303+ [cells]** \| \| **Lin2-CD1c+CD141+ [cells]** \| \| **Lin2-CD1c+CD141+CD303+ [cells]** \| \| **Lin2-CD141+CD303+ [cells]** \| \| **Lin2-CD1c+CD303+ [cells]** \| \| **mDC2/mDC1 ratio CD141+/CD1c+** \| \| **mDC2/pDC ratio CD141+/CD303+** \| \| **mDC1/pDC ratio CD1c/CD303** \| | \| 0,340 \| \| --- \| \| -0,030 \| \| 0,273 \| \| -0,030 \| \| -0,153 \| \| 0,224 \| \| -0,153 \| \| 0,443 \| \| -0,036 \| \| 0,194 \| \| -0,085 \| \| 0,170 \| \| 0,237 \| \| 0,170 \| \| 0,364 \| \| -0,048 \| \| 0,218 \| \| -0,048 \| \| 0,042 \| \| 0,218 \| \| 0,042 \| \| 0,310 \| \| 0,115 \| \| -0,522 \| | \| 0,333 \| \| --- \| \| 0,938 \| \| 0,440 \| \| 0,938 \| \| 0,672 \| \| 0,528 \| \| 0,672 \| \| 0,198 \| \| 0,925 \| \| 0,588 \| \| 0,817 \| \| 0,633 \| \| 0,506 \| \| 0,633 \| \| 0,298 \| \| 0,898 \| \| 0,541 \| \| 0,898 \| \| 0,910 \| \| 0,541 \| \| 0,910 \| \| 0,379 \| \| 0,750 \| \| 0,124 \| | \| 0,244 \| \| --- \| \| -0,181 \| \| -0,028 \| \| -0,181 \| \| 0,408 \| \| -0,062 \| \| 0,408 \| \| **0,580** \| \| -0,139 \| \| 0,286 \| \| -0,153 \| \| **0,719** \| \| 0,335 \| \| **0,719** \| \| **0,566** \| \| -0,104 \| \| 0,328 \| \| -0,048 \| \| **0,671** \| \| 0,328 \| \| **0,671** \| \| **0,531** \| \| 0,335 \| \| -0,321 \| | \| 0,443 \| \| --- \| \| 0,573 \| \| 0,933 \| \| 0,573 \| \| 0,189 \| \| 0,851 \| \| 0,189 \| \| **0,052** \| \| 0,667 \| \| 0,366 \| \| 0,635 \| \| **0,010** \| \| 0,286 \| \| **0,010** \| \| **0,059** \| \| 0,749 \| \| 0,297 \| \| 0,886 \| \| **0,020** \| \| 0,297 \| \| **0,020** \| \| **0,079** \| \| 0,286 \| \| 0,308 \| | \| -0,033 \| \| --- \| \| **-0,616** \| \| -0,317 \| \| **-0,616** \| \| 0,142 \| \| -0,366 \| \| 0,142 \| \| 0,116 \| \| **-0,716** \| \| -0,050 \| \| **-0,716** \| \| 0,343 \| \| -0,100 \| \| 0,343 \| \| 0,116 \| \| **-0,650** \| \| 0,050 \| \| **-0,600** \| \| 0,283 \| \| 0,050 \| \| 0,283 \| \| **0,716** \| \| 0,483 \| \| -0,416 \| | \| 0,948 \| \| --- \| \| **0,085** \| \| 0,401 \| \| **0,085** \| \| 0,717 \| \| 0,336 \| \| 0,717 \| \| 0,775 \| \| **0,036** \| \| 0,911 \| \| **0,036** \| \| 0,364 \| \| 0,809 \| \| 0,364 \| \| 0,775 \| \| **0,066** \| \| 0,911 \| \| **0,096** \| \| 0,462 \| \| 0,911 \| \| 0,462 \| \| **0,036** \| \| 0,193 \| \| 0,269 \| | \| -0,500 \| \| --- \| \| 0,133 \| \| 0,033 \| \| 0,133 \| \| 0,133 \| \| 0,100 \| \| 0,133 \| \| -0,116 \| \| 0,400 \| \| 0,116 \| \| 0,400 \| \| 0,000 \| \| 0,116 \| \| 0,000 \| \| -0,083 \| \| 0,383 \| \| 0,116 \| \| 0,300 \| \| 0,033 \| \| 0,116 \| \| 0,033 \| \| -0,366 \| \| -0,266 \| \| 0,150 \| | \| 0,177 \| \| --- \| \| 0,743 \| \| 0,937 \| \| 0,743 \| \| 0,743 \| \| 0,809 \| \| 0,743 \| \| 0,775 \| \| 0,291 \| \| 0,775 \| \| 0,291 \| \| 1,000 \| \| 0,775 \| \| 1,000 \| \| 0,843 \| \| 0,312 \| \| 0,775 \| \| 0,436 \| \| 0,948 \| \| 0,775 \| \| 0,948 \| \| 0,336 \| \| 0,493 \| \| 0,708 \| |

| **1 year of treatment (T2)** | **TSH [mIU/l]** | | **fT4 [ng/dl]** | | **fT3 [ng/l]** | | **TRAb [IU/l]** | |
| --- | --- | --- | --- | --- | --- | --- | --- | --- |
|  | **r** | **p** | **r** | **p** | **r** | **p** | **r** | **p** |
| \| **CD141+ [% of Lin2- PBMC]** \| \| --- \| \| **CD1c+ [% of Lin2- PBMC]** \| \| **CD303+ [% of Lin2- PBMC]** \| \| **CD1c+CD141+ [% of Lin2- PBMC]** \| \| **CD1c+CD141+CD303+ [% of Lin2- PBMC]** \| \| **CD141+CD303+ [% of Lineage- PBMC]** \| \| **CD1c+CD303+ [% of Lin2- PBMC]** \| \| **Lin2-CD141+ [% of PBMC]** \| \| **Lin2-CD1c+ [% of PBMC]** \| \| **Lin2-CD303+ [% of PBMC]** \| \| **Lin2-CD1c+CD141+ [% of PBMC]** \| \| **Lin2-CD1c+CD141+CD303+ [% of PBMC]** \| \| **Lin2-CD141+CD303+ [% of PBMC]** \| \| **Lin2-CD1c+CD303+ [% of PBMC]** \| \| **Lin2-CD141+ [cells]** \| \| **Lin2-CD1c+ [cells]** \| \| **Lin2-CD303+ [cells]** \| \| **Lin2-CD1c+CD141+ [cells]** \| \| **Lin2-CD1c+CD141+CD303+ [cells]** \| \| **Lin2-CD141+CD303+ [cells]** \| \| **Lin2-CD1c+CD303+ [cells]** \| \| **mDC2/mDC1 ratio CD141+/CD1c+** \| \| **mDC2/pDC ratio CD141+/CD303+** \| \| **mDC1/pDC ratio CD1c/CD303** \| | \| -0,200 \| \| --- \| \| -0,300 \| \| 0,300 \| \| -0,300 \| \| -0,200 \| \| 0,300 \| \| -0,200 \| \| -0,200 \| \| -0,500 \| \| -0,300 \| \| -0,500 \| \| -0,200 \| \| -0,300 \| \| -0,200 \| \| -0,200 \| \| -0,100 \| \| 0,100 \| \| -0,100 \| \| -0,200 \| \| -0,300 \| \| -0,200 \| \| 0,600 \| \| -0,300 \| \| -0,500 \| | \| 0,783 \| \| --- \| \| 0,683 \| \| 0,683 \| \| 0,683 \| \| 0,783 \| \| 0,683 \| \| 0,783 \| \| 0,783 \| \| 0,450 \| \| 0,683 \| \| 0,450 \| \| 0,783 \| \| 0,683 \| \| 0,783 \| \| 0,783 \| \| 0,950 \| \| 0,950 \| \| 0,950 \| \| 0,783 \| \| 0,683 \| \| 0,783 \| \| 0,350 \| \| 0,683 \| \| 0,450 \| | \| 0,200 \| \| --- \| \| 0,800 \| \| -0,400 \| \| 0,800 \| \| -0,600 \| \| -0,400 \| \| -0,600 \| \| 0,200 \| \| 0,400 \| \| 0,200 \| \| 0,400 \| \| -0,600 \| \| 0,200 \| \| -0,600 \| \| 0,200 \| \| 0,200 \| \| -0,400 \| \| 0,200 \| \| -0,600 \| \| 0,200 \| \| -0,600 \| \| -0,800 \| \| 0,800 \| \| 0,800 \| | \| 0,916 \| \| --- \| \| 0,333 \| \| 0,750 \| \| 0,333 \| \| 0,416 \| \| 0,750 \| \| 0,416 \| \| 0,916 \| \| 0,750 \| \| 0,916 \| \| 0,750 \| \| 0,416 \| \| 0,916 \| \| 0,416 \| \| 0,916 \| \| 0,916 \| \| 0,750 \| \| 0,916 \| \| 0,416 \| \| 0,916 \| \| 0,416 \| \| 0,333 \| \| 0,333 \| \| 0,333 \| | \| 0,500 \| \| --- \| \| 1,000 \| \| -0,500 \| \| 1,000 \| \| -0,500 \| \| -0,500 \| \| -0,500 \| \| 0,500 \| \| 0,500 \| \| 0,500 \| \| 0,500 \| \| -0,500 \| \| 0,500 \| \| -0,500 \| \| 0,500 \| \| 0,500 \| \| -0,500 \| \| 0,500 \| \| -0,500 \| \| 0,500 \| \| -0,500 \| \| -0,500 \| \| 1,000 \| \| 0,500 \| | \| 1,000 \| \| --- \| \| 0,333 \| \| 1,000 \| \| 0,333 \| \| 1,000 \| \| 1,000 \| \| 1,000 \| \| 1,000 \| \| 1,000 \| \| 1,000 \| \| 1,000 \| \| 1,000 \| \| 1,000 \| \| 1,000 \| \| 1,000 \| \| 1,000 \| \| 1,000 \| \| 1,000 \| \| 1,000 \| \| 1,000 \| \| 1,000 \| \| 1,000 \| \| 0,333 \| \| 1,000 \| | \| -0,542 \| \| --- \| \| -0,771 \| \| -0,085 \| \| -0,771 \| \| 0,485 \| \| -0,142 \| \| 0,485 \| \| -0,600 \| \| -0,371 \| \| -0,542 \| \| -0,371 \| \| 0,428 \| \| -0,542 \| \| 0,428 \| \| -0,600 \| \| **-0,885** \| \| -0,600 \| \| **-0,885** \| \| 0,428 \| \| -0,542 \| \| 0,428 \| \| 0,428 \| \| -0,600 \| \| -0,428 \| | \| 0,297 \| \| --- \| \| 0,102 \| \| 0,919 \| \| 0,102 \| \| 0,355 \| \| 0,802 \| \| 0,355 \| \| 0,241 \| \| 0,497 \| \| 0,297 \| \| 0,497 \| \| 0,419 \| \| 0,297 \| \| 0,419 \| \| 0,241 \| \| **0,033** \| \| 0,241 \| \| **0,033** \| \| 0,419 \| \| 0,297 \| \| 0,419 \| \| 0,419 \| \| 0,241 \| \| 0,419 \| |
