# Supplementary figures and images for: Evaluating the Role of Circulating Dendritic Cells in Methimazole-Treated Pediatric Graves’ Disease Patients
Source: Genes (Basel). 2021 Jan 26;12(2):164. doi: 10.3390/genes12020164 (PMC7911035; doi:10.3390/genes12020164)

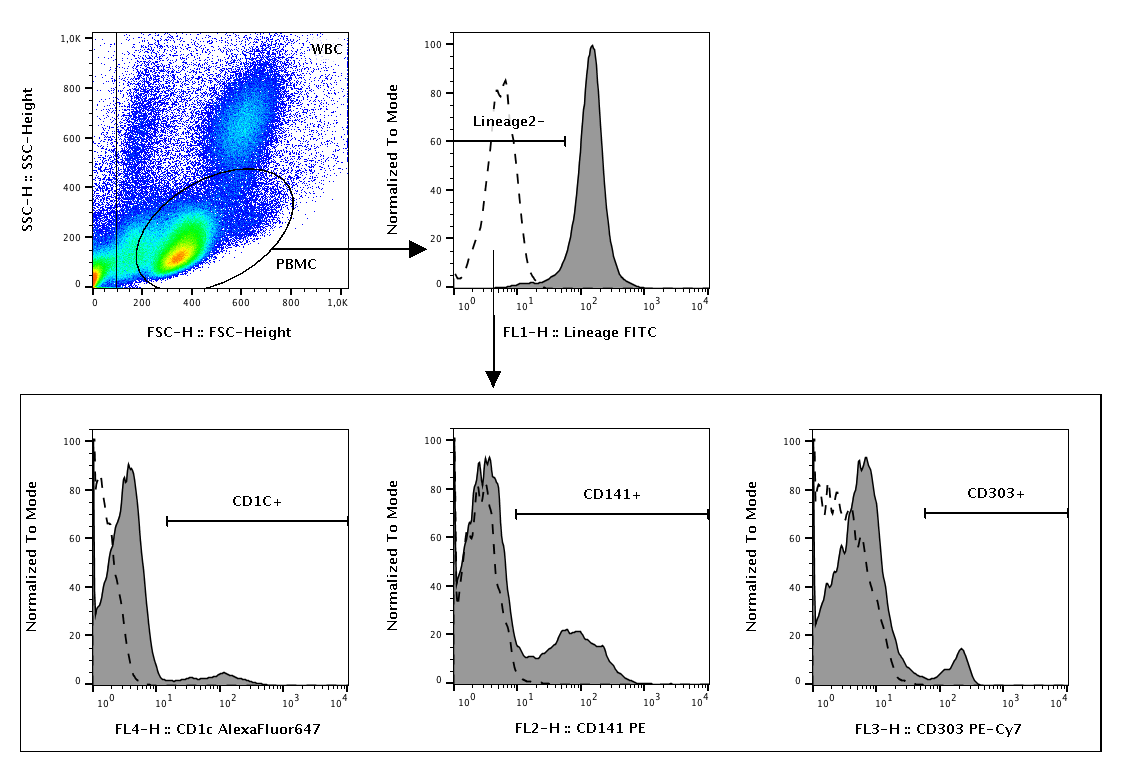

Supplement: Supplementary file 1 [file genes-12-00164-s001.zip › supplementary/Supp. Fig. 1.tiff]
